# Extending the Shelf-Life of Live Clams, *Venerupis corrugata*—Important Aspects of Current Packaging and Advances in Modified Atmosphere Packaging

**DOI:** 10.3390/foods14091629

**Published:** 2025-05-05

**Authors:** Cintia Borghetti Goes, Susana Teixeira, Cristina Mena, Fátima Silva, Andreia Cruz, Inês Basílio, Maria Conceição Hogg, Morten Sivertsvik, Paula Teixeira, Fátima Poças

**Affiliations:** 1 Universidade Católica Portuguesa, CBQF—Centro de Biotecnologia e Química Fina—Laboratório Associado, Escola Superior de Biotecnologia, Rua Diogo Botelho 1327, 4169-005 Porto, Portugal; cgoes@ucp.pt (C.B.G.); mhogg@ucp.pt (M.C.H.); pcteixeira@ucp.pt (P.T.); 2 Escola Superior de Biotecnologia, Centre for Food Quality and Safety, Universidade Católica Portuguesa, Rua Diogo Botelho 1327, 4169-005 Porto, Portugal; steixeira@ucp.pt (S.T.); cmena@ucp.pt (C.M.); mfbsilva@ucp.pt (F.S.); 3 Oceano Fresco S.A., Porto da Nazaré, 2450-075 Nazaré, Portugal; andreia.cruz@oceano-fresco.pt (A.C.); ines.basilio@oceano-fresco.pt (I.B.); 4 Department of Processing Technology, Nofima AS, Richard Johnsen gt 4, 4021 Stavanger, Norway; morten.sivertsvik@nofima.no

**Keywords:** bivalve molluscs, pullet carpet shells, temperature storage, survival percentage, quality, net bags, tightness packaging, MAP

## Abstract

*Venerupis corrugata* (pullet carpet shell) is a premium native clam species in Portugal. This species is highly perishable, typically sold live within 3 or 4 days, posing a significant risk of loss. Therefore, efforts to extend its shelf-life are relevant. The impact of the storage temperature (3, 5, 8 and 12 °C) on clams in plastic net bags and the effect of modified atmosphere packaging (MAP) were investigated. The survival percentage and microbiological and chemical parameters were evaluated, as well as sensory characteristics. The survival percentage and sensory aspects results indicate that the longest time with 95% live clams was observed at 5 °C and 8 °C, but lower temperatures (3 and 5 °C) have lower death rates after the threshold. In the MAP tests, the clams were kept closed due to confinement in plastic trays applying a vacuum, before gas flushing that drew the lid film over the clams. However, a negative effect of CO_2_ was observed for clams, with lower survival when packaged in 30% CO_2_. The shelf-life increased by only 1–2 days under >70% O_2_ with no CO_2_. These results show that this species is very sensitive, and MAP is not commercially effective for this application.

## 1. Introduction

*Venerupis corrugata* or pullet carpet shell is a bivalve mollusc species classified in the family Veneridae. Its shell is typically thin and elongated, with concentric lines stronger on its external surface. The maximum size of shells is approximately 5 cm, and 3.8 cm is the minimum dimension for capture in Portugal [1]. This species has some differences in appearance compared with related clam species, such as the united siphons along the entire length of the animal except in the terminal zone [2]. The pullet carpet shell inhabits sandy or muddy substrates in shallow waters. It is a European native species, often found in the coastal areas of the northeastern Atlantic Ocean, including the North Sea and the Mediterranean Sea.

Animal-based protein derived from clams is a highly sustainable source of protein as these are low-trophic-level species. Consumers appreciate clams due to their taste. Additionally, there are also nutritional benefits associated with their consumption as they are rich in vitamins (especially B12) and essential minerals (iodine, selenium and calcium), low in fat and a good source of omega-3 fatty acids with well-established health benefits [3].

The demand for European native species is evident within producers and consumers due to their high gastronomic, nutritional and higher economic value (about 3× higher in price per kg than the invasive species) [4,5]. Despite a EUR 6.3 billion+ global clam market [6] that is already established, a few gaps along the value-chain exist that need to be overcome to increase the shellfish industry standards and to support the production, packaging and supply of European autochthonous species such as *V. corrugata*. Large-scale production and supply capacity is lacking. New technologies and concepts of products to extend their shelf-life are needed to reach new markets in a sustainable way.

After being removed from the water, fresh clams, *V. corrugata*, present a good quality and are usually sold within 3–4 days under refrigeration conditions. The Codex Alimentarius Commission standard for live and raw bivalve molluscs CXS 292-2008 considers that “Bivalves must be alive when sold”. It sets a tolerance of 5% maximum damage or dead clams by count [7]. However, other commercial standards have been proposed, such as a minimum of 80% viability [8,9]. Damage to shells mostly leads to death, and the cause is directly associated with incorrect handling before retailing [10,11]. In addition to shell damage, the clam survival time is related to other factors which can impact the days of life, particularly temperature and maintaining the shell as closed to avoid the exudation of intravalvular fluids [12].

The quality of the clams is highly dependent on the water quality where they are captured [13]. Therefore, the authorities monitor and classify the water of the production areas and the bivalve molluscs. European regulations establish limits for *Escherichia coli*, marine toxins such as lipophilic Diarrhetic Shellfish Poisoning (DSP), Paralytic Shellfish Poisoning (PSP) and Amnesic Shellfish Poisoning (ASP), metals and Polycyclic Aromatic hydrocarbons (PAHs) and the production zones are classified accordingly. Different zones determine whether the bivalve is suitable for direct sales or needs an additional process of depuration before commercialisation. Clams from Zone A can be sold directly, from Zone B must go through a depuration process and clams from Zone C, generally, must be transferred to a class A or B area for at least two months followed by a depuration process or be industrially transformed/ processed [14,15].

The temperature must be controlled from depuration throughout the packaging process, distribution and display at the selling point and then by the consumers. Inadequate transportation modes were associated with low survival rates and serious quality loss problems in fresh aquatic products [11]. Anacleto et al. [16,17] studied the effect of depuration and two different temperatures of transport (4 and 22 °C) in physiological and microbiological responses of pullet carpet shell (*V. corrugata*) and manila clam (*Ruditapes philippinarum*). Depuration positively impacted all conditions and species, reducing the bacterial load. However, the transportation temperature had a greater impact on mortality, revealing the sensitivity of the European native species, *V. corrugata*. Considering a 50% survival rate, *R. philippinarum* exhibited a 10-day increase in lifespan when transported at 4 °C compared to 22 °C, while *V. corrugata* only showed a 2-day increase. Bi et al. [18] examined the survival rate and the physiological responses of *R. philippinarum* clams transported for 3 days with and without water, at two temperatures, 4 °C and 15 °C. The results indicated that the anhydrous transportation at 4 °C was the most suitable method.

Fresh live clams are generally sold in plastic polypropylene (PP), polyethylene (PE) or polyamide (PA) net bags and they are displayed on the market with or without placing the bags on ice. Although light, versatile and easy to use, this plastic net has the disadvantage of easy shell breakage during handling and a lack of hygiene due to the continuous drainage of fluid from the mollusc cavity [12]. The key is the balance between tightness required to keep the clams closed and looseness to avoid mechanical damage to the shells.

Modified atmosphere packaging (MAP) and vacuum packaging (VP) are important technologies that have long been used for shellfish and fish products to reduce the respiratory rate and metabolic processes, inhibit the growth of microorganisms during storage and increase the shelf-life [12,19,20,21]. The important parameters to increase the shelf-life of bivalves with MAP are the composition of the gaseous mixture, the packaging system (material and geometry) and the temperature control. These determine the ratio between the gas and the product, the barrier to gas permeation and the ability to maintain the right gas composition during storage. As an example, Pastoriza and Bernárdez [12] studied MAP with live mussels and noted that they can have a shelf-life of between 5 and 15 days in MAP depending on the treatments before packaging, the mixture of gases and the refrigeration temperature controlled during the storage periods to keep the bivalves in a lethargic state.

Carbon dioxide is widely used in MAP due to its antimicrobial effects. Dissolving CO_2_ in the product results in an increased lag phase and a slower rate of microbial growth during the logarithmic phase [21]. Some studies with mussels (*Mytilus galloprovincialis*) have focused on the effect of different gas mixtures in MAP to extend the survival percentage [10]. They observed that the mortality of mussels increased with the presence of CO_2_ gas in the gas composition. In addition to the indication of a low CO_2_ concentration, there is a recommendation for gaseous mixtures with a high oxygen concentration, above 50%, which improve the survival time of live mussels compared to storage in air. Ratnawati et al. [9] showed that it was possible to extend nine days of life span for blue mussels (*Mytilus edulis*) in MAP with 40% CO_2_: 60% O_2_ stored at 4 °C, considering mortality < 20%. A modified atmosphere with a high oxygen concentration (75–85% O_2_) extended the shelf-life of mussels by up to 8 days, considering a storage temperature controlled at a maximum of 2 °C [8].

Different species of bivalves may react differently to the gas mixture content, which justifies studies with other species, in particular, those of commercial importance. The scientific literature contains many shelf-life studies for bivalve molluscs [21,22], mainly mussels [8,9] and oysters [23,24]. The effect of a gas mixture with a high concentration of O_2_ in MAP (70% O_2_: 30% N_2_) was studied in live clams (*Ruditapes decussatus*) stored at 6.0 ± 0.7 °C and resulted in a shelf-life of six days with good sensory characteristics [25]. However, studies focusing on *V. corrugata* species as a food are not reported. This species is considered premium in Europe and a good candidate for aquaculture. Therefore, increasing its shelf-life is of major interest for commercial value and economic benefits.

The present study aimed to evaluate the effect of two strategies to increase the time for 95% survival of *V. corrugata* clams: (a) the controlled storage temperature of clams packaged in plastic net bags and (b) gas composition and packaging conditions of MAP during storage at 3 °C.

## 2. Materials and Methods

### 2.1. Clam Samples

Live clams (*V. corrugata*) were collected from the west Atlantic coast of Portugal (shell length 3.8–5.3 cm, 13.0 ± 3.0 g), respecting the minimum size for sale. The clams were locally supplied within 24–48 h of depuration and used on the same day for the tests. All samples were transported to the lab packaged in 1 kg plastic net bags and under refrigeration (3 °C).

**Figure 1:**
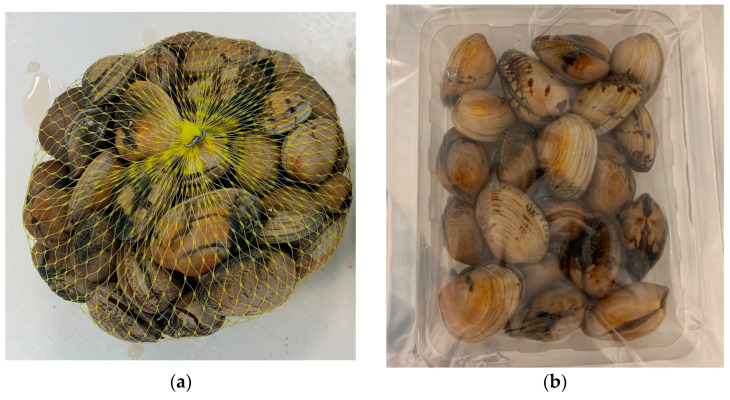
Clams’ packaging: (**a**) net bag used in temperature tests; (**b**) tray and lid used in MAP tests.

This study used six independent lots of clams (tests T1–T6) collected between May and July 2023. Each lot, with clams packaged in 1 kg plastic net bags (Figure 1a), was evaluated for their chemical composition, microbiological quality, percentage of survival and sensory analysis. Exudated liquid quantification and headspace gases analysis were also performed.

### 2.2. Effect of Storage Temperature

The clams were stored in refrigerated incubators at 3 °C ± 1 °C and 5 °C ± 2 °C (MIR253, Sanyo, Osaka, Japan) and at 8 °C ± 1 °C (MIR253, Sanyo, Osaka, Japan) and 12 °C ± 2 °C (FitoClima Aralab 1200, Rio de Mouro, Portugal). The samples were evaluated at 0, 3, 4, 5 and 6 days of storage. The temperatures were chosen to cover the storage and transport range of other refrigerated products (2–7 °C) and to extrapolate to higher temperatures, closer to the region’s seawater, but it is currently considered unsuitable for refrigerated transport (7–14 °C).

### 2.3. Impact of MAP

The clams were packaged in thermoformed monolayer polypropylene (PP) trays (148 × 117 × 34 mm) with a lid multilayer barrier film composed by polyamide, ethylene vinyl alcohol and polyethylene (PA/EVOH/PE120 µm) (oxygen transmission rate (OTR): <1.5 cm^3^/m^2^.24 h at 23 °C/50% RH, water vapor transmission rate (WVTR): <7 g/m^2^.24 h at 38 °C/90% RH) (Figure 1b).

Preliminary tests were carried out to define the packaging geometry in relation to the number of clams and the parameters of the MAP equipment vacuum and gas flushing pressure. The vacuum level was set at 50 mbar to remove the headspace air and to draw the lidding film over the clams without mechanical damage. The gas flushing pressure affects the gas mixture amount available inside the package, and two levels were tested: 120 mbar and 150 mbar. A free-standing chamber MAP machine with a gas flushing system (Multivac Gastrovac, Carnaxide, Portugal) was used. All gases were food-grade from Gasin/Air Product (Spain). Table 1 presents the operational parameters of MAP equipment and the gas mixture (G1–G3) used in each condition.

Twenty (20) clams were packed per tray in both tests. These corresponded to 280–345 g clams’ weights depending on the clam’s size. Samples were stored at 3 °C ± 1 °C and evaluated at 0, 3 and 6 days (5 replicates in each sampling time) and for tests with gas mixture G1 (70% O_2_: 30% CO_2_) and gas mixture G2 (70% O_2_: 30% N_2_) conditions, and at 0, 2, 5, 6, 7, 8 and 9 days (2 replicates in each sampling time) for tests with G3 (90% O_2_: 10% N_2_) condition.

### 2.4. Chemical Analysis

The moisture content was determined by oven drying (Memmert ULE 600, Porto Salvo, Portugal) according to NP 2282:2009. In total, 10 g clam flesh was mixed with 25 g of sand and dried at 105 ± 2 °C for 3 h with a repeated drying period of 1 h until constant weight.

The pH was measured at 20 ± 2 °C using a pH meter (Crison GLP 22+, Barcelona, Spain) and a combined pH electrode (Crison 50 14T) in a sample suspension of 10 g of homogenised clam with 100 mL deionised water.

The total volatile basic nitrogen content (TVB-N) was determined according to NP 2930:2009, Conway microdiffusion method, using 1 mL filtrate extract-obtained mixing, over 2 min in a Turrax homogeniser and 50 g flesh sample with 100 mL of trichloroacetic acid 0.5% m/v solution. The volatile bases were released from the filtrate, alkalised with sodium carbonate for 90 min at 40 °C in an oven, collected in a boric acid 1% m/v solution and titrated with HCl 0.1 mol/L standard solution.

### 2.5. Microbiological Analysis

Microbiological analyses were performed on the day of sampling for all tests and during the shelf-life for MAP tests using standardised methods. An initial suspension was prepared with 10 g of clam flesh in 90 mL of Ringer’s solution. After homogenisation in the stomacher, appropriate decimal dilutions were also prepared in sterile Ringer’s solution to total viable count (TVC) at 7 °C and 25 °C following the standard methods of the International Organization for Standardization [26,27], respectively, but using plate count agar (PCA) with 1% NaCl added to support the growth of the salt-requiring bacteria. Plates were incubated at 25 ± 1 °C for 5 days for TVC at 25 °C and 7 ± 1 °C for 10 days for TVC at 7 °C. The results are expressed as the logarithm of colony-forming units per gram (log cfu/g) or due comparison normalised values expressed in log N/N0.

### 2.6. Survival Percentage

The percentage of live clams was evaluated by observing the closure of valves while stimulating them by hand, a method reported by Gonçalves et al. [25] and used also by Bi et al. [18]. On each sampling day, individual clams were observed and counted: the total number of clams, clams opened, clams closed, damaged shells and live and dead clams. The individual was considered alive if the shell valve closed or the body moved. The survival percentage was calculated according to Equation (1).

(1) $$Live\;clams\;\left(\%\right)=\frac{\left(Nº\;live\;individuals+damaged\;alive\right)}{Nº\;total\;clams\;evaluated}\times 100$$

### 2.7. Sensory Analysis

The freshness of live clams was assessed by a panel of five non-expert members. The sensory parameters of appearance and odour using the scale and descriptors mentioned in Table 2 were evaluated. The score of 1 or below, on a scale 0 to 3, was considered unacceptable.

### 2.8. Exudate

The exudate was quantified by weighing the residual liquid in the packaging at each sampling point and expressed in grams. This analysis was completed only in MAP tests.

### 2.9. Headspace Gases Analysis

The gas composition (% CO_2_ and O_2_) inside the packages was measured with a headspace gas analyser (CheckMate 3^®^, Dansensor, Neuwied Germany).

### 2.10. Statistical Analysis

The results of initial characterisation of the clams and comparisons between more than two conditions were statistically analysed by one-way analysis of variance (ANOVA) conducted using IBM SPSS Statistics Version 30 (SPSS Inc., Chicago, IL, USA). Post hoc comparison Tukey HSD test was used to determine significant differences. A *p*-value < 0.05 was considered to indicate statistical significance. Comparison between only two conditions was analysed by *t*-test.

## 3. Results

### 3.1. Variability of the Samples

The initial quality of the clams used in each test was screened through microbiological and physical–chemical analyses. The average results are presented in Table 3.

The values for the pH ranged between 6.4 and 6.6; the moisture ranged between 82.4% and 90.4%; the TVB-N from <0.6 to 22.2 mg N/100 g; TVC at 7 °C ranged from 1 to 4 log cfu/g; and TVC at 25 °C ranged from 3.3 to 4.8 log cfu/g. A wide range of individual weights was observed, from 6.4 g to 16.4 g, depending on the flesh and the shell size. The flesh of clams can represent between 2% and 17% of their total weight.

In terms of TVB-N, the clams of tests T2, T5 and T6 (Table 3), used in repeated tests to access the effect of the temperature, had much higher initial values than the other batches, although they were still below the freshness threshold of 30 mg N/100 g for fish [14,15,28]. The measurements, as the method described, were performed on 50 g of clam flesh sampled from each 1 kg of clams received packaged from the supplier. The high intra-individual variability could result in a single high TVB-N individual giving a higher average for the whole sample. As this could be the reason for the high values reported in batches T2, T5 and T6, these batches’ replicates were not considered in the evaluation of the results.

### 3.2. Impact of Temperature

**Figure 2:**
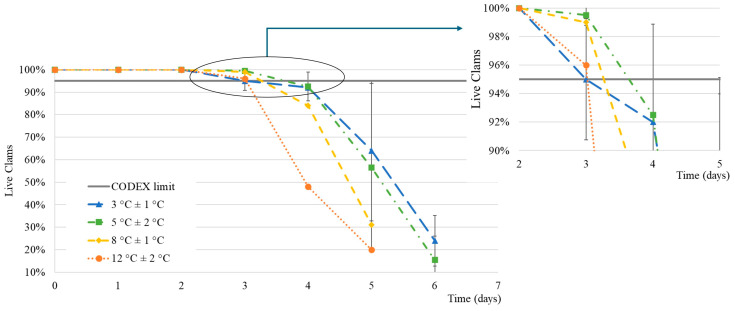
Survival percentage (%) of clams stored at different temperatures. Detail of the time for CODEX limit (5% of non-live clams). Average ± standard deviation.

The survival percentage of live clams stored at different temperatures was recorded over time (Figure 2).

Despite the high variability expected in this type of study (see Section 3.1 regarding the variability inter-batch), the results indicate that temperature impacts the clams’ survival. The longest time for 5% death was observed for intermediate temperatures (5 and 8 °C). However, to reach a 10% death percentage, clams stored at 3 and 5 °C took the longest time, 5.5 and 5.3 days, respectively, whereas under 8 °C, this reduced to 4.6 days. At 12 °C, after just 4 days, 50% of the clams were dead.

A sensory analysis showed that the clams stored at 8 °C and 12 °C lost freshness and were considered unacceptable on the 4th day (Figure S1a). It was also observed that the number of open shells at the time of the sensory analysis increased with time, which suggests that it is related to the percentage of death. The shells are interconnected to the adductor muscle and its contraction closes the shell—when the bivalve dies, the muscle can no longer contract the ligament and the shells open. As a result, the clams lose the intravalvular fluid that aids in the breathing process as this is normally carried out through the oxygen dissolved in the water that passes through the gills [29].

The clams stored at 3 °C showed some superficial dehydration of the shells, even though the storage was at 80% RH with air circulation. To minimise this effect, the packages were turned over every day. The clams stored at 8 and 12 °C began to exude mucus, like drool, after the fourth day onwards. This effect was not observed in the clams stored at lower temperatures.

The odour was considered unacceptable on the 6th day when stored at 3 °C and 5 °C and on the 4th day when stored at 8 °C and 12 °C, as shown in the Supplementary Information (Figure S1b). It was observed that the odour is a very important parameter in the sensory analysis of this type of product since the loss of freshness is quickly noticed in the samples on the same evaluation day meaning that the detection and counting of dead clams were identified. Additionally, 24 h after this detection, the odour was assessed as unacceptable in all samples and the number of dead clams quickly increased (Figure 2).

### 3.3. Impact of MAP

As mentioned previously, after preliminary tests, the vacuum (50 mbar) and the gas flushing pressure (120–150 mbar) were defined aiming at obtaining the maximum gas mixture available, keeping the clams confined by the action of the initial vacuum on drawing the lidding film over them. The gases’ concentrations were selected based on the few studies available for bivalves such as mussels. The impact of the presence of CO_2_ in mixtures of a high O_2_ level was studied, as well as the impact of increasing the O_2_ level in the absence of CO_2_ in the initial mixture.

#### 3.3.1. Effect of CO_2_

The impact of carbon dioxide on the percentage of live clams, exudation, chemical and microbiological analysis over time is shown in Table 4. The G2a condition (70% O_2_: 30% N_2_, 150 mbar) showed a higher survival percentage for a longer storage time without CO_2_. It seems that CO_2_ has a detrimental effect on keeping clams alive and the higher pressure suggests greater gas availability in the packaging headspace.

For all conditions, the exudate of the clams increased with the storage time, as expected. However, the large variability between replicates meant that any significant difference between the conditions could not be statistically validated.

The effect of the carbon dioxide concentrations for preserving seafood products has been highlighted in the literature [20,21]. In the present case, the values for the TVC change at 7 °C increase ca 2 log up to day 3, and then the growth rate slows down up to day 6, with a trend observed for lower counts for the samples packaged under CO_2_. The results for the TVC change at 25 °C are stable until the 3rd day and then increase ca 1 log up to day 6 (Table 4). Therefore, the results indicate that despite this small advantage in slowing down spoilage, gas mixtures with carbon dioxide do not aid the survival of clams.

The sensory evaluation results were presented in Table 5. The appearance of the samples received the maximum marks (score 3) until the 6th day. Regarding odour, there was a loss of freshness detected, but the samples were considered acceptable (Score > 1) until the 6th day.

Regarding the chemical analyses, the pH and moisture values did not show significant differences between the conditions over time. The average for pH was 6.7 ± 0.1 and for moisture was 88.6 ± 1.4%. A lower pH value was expected for samples under the G1 condition, consistent with CO_2_ solubility in the tissues according to Equation (2), as reported before for seafood [30].

(2) $$CO_{2}+H_{2}O↔H_{2}CO_{3}↔HCO_{3}^{-}+H^{+}$$

Although reported in post-mortem processes and degradation in meat products [31], a correlation between pH and TVB-N was not observed in this study, possibly due to its short duration. TVB-N increased over time, with values similar for all conditions until the 3rd day. On the 6th day, the lowest values were detected when clams were stored in the presence of CO_2_ with 11.40 ± 0.04 mg N/100 g. The highest TVB-N was detected for the G2b condition corresponding to the highest number of clams alive. Nevertheless, the maximum value obtained for the 6th day was 18.06 ± 0.56 mg N/100 g, which, if much lower than the reference for fish, considered 30 mg N/100 g as the freshness threshold [14,15,28]. All average values are presented in Table 4.

A possible cause of the early death of *V. corrugata* when in the presence of carbon dioxide may be the acceleration of the metabolism leading to the increase in the respiration rate of this species, as reported for other molluscs like *Mercenaria mercenaria* [32]. The same was reported for mussels, with a remarkable survival for mixtures of low levels of CO_2_ [10]. It was reinforced that the high solubility of CO_2_, that can penetrate even closed shells and consequently acidify the medium, was considered more harmful to maintaining the clam’s life than contributing with a bacteriostatic effect.

Since the respiration process generates CO_2_, and this level being naturally formed may be enough to increase the death rate, its elimination could be relevant. A toxicity limit of <1% or the complete elimination of this carbon dioxide is cited by [12]. Therefore, active packaging systems able to remove CO_2_ from the headspace below quickly would be needed.

#### 3.3.2. Impact of MAP with High Oxygen

**Figure 3:**
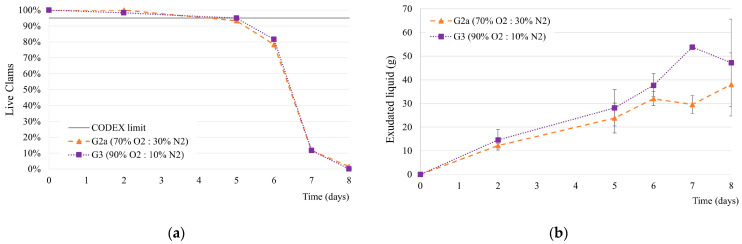
Survival percentage (**a**) and amount of liquid exudate (**b**) for live clams under MAP conditions (G2a, G3) over time. Average ± standard deviation.

In addition to the indication of a low CO_2_ concentration, gas mixtures with a high oxygen concentration above 50%, were reported to increase the survival time of live mussels compared to normal air [10]. This needs verification for *V. corrugata* because no studies were reported for this species, and different species of bivalves may react differently to a high oxygen content. The impact of the high-oxygen-concentration atmospheres G2a (70% O_2_: 30% N_2_) and G3 (90% O_2_: 10% N_2_) were studied in this experiment. No difference was observed between the survival percentages for both gas mixtures of a higher oxygen concentration tested (Figure 3a). In both cases, the clams’ 95% alive limit was maintained up to day 5 and similar death rates were observed afterwards.

The clam’s exudate increased during the initial storage time and seemed to show an unclear trend to stabilise after day 6. Despite the high variability between replicates, a relatively higher exudate amount was observed for the clams packaged in the higher oxygen concentration (Figure 3b).

The results for the concentration of gases in the headspace show a decrease in the oxygen from 63.6 ± 3.5% to 48.2 ± 2.3% and from 90.3 ± 3.4% to 73.6 ± 1.8% after 9 days, respectively, for clams in the mixtures G2a and G3. The carbon dioxide increased on the last day of the clam’s evaluation (day 9) by 3% and 7% for the clams in mixtures G2a and G3. The plots are included in the Supplementary Information (Figure S2).

The chemical assessment values, presented in the Supplementary Information (Figure S3), show no significant difference in the results obtained in the G2a and G3 conditions of pH and moisture. The TVB-N results increased over time for both conditions, with a markedly higher rate after day 5. A final average value of 19.2 mg N/ 100 g was achieved on the 8th day.

The sensory analysis of the clams packaged in the G2a and G3 conditions (Figure S4) did not show significant differences, and the higher oxygen concentration (90% O_2:_ 10% N_2_) did not result in a longer shelf-life than the lower concentration tested. Although the clams’ survival rate dropped after the 5th day, they maintained a good appearance (score 2) until day 8. The fresh odour changed over time, becoming unacceptable (score 0) for both samples on the 8th day with 100% dead clams. The odour of the clams under the MAP condition G3 was slightly better than the condition with G2a (score 1 on 7th day).

**Figure 4:**
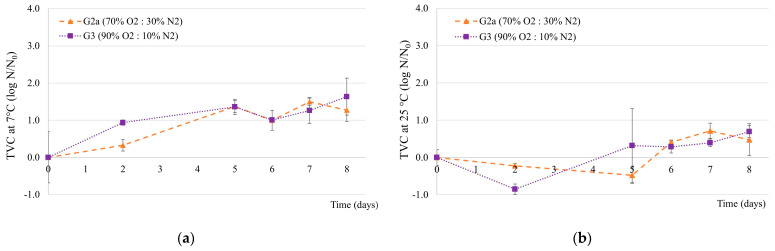
Changes in total microorganisms count at 7 °C (**a**) and 25 °C (**b**) under MAP conditions (G2a, G3) over time. Normalised averages ± standard deviation.

The total viable count changes during storage at 7 °C and 25 °C under the two tested conditions (G2a and G3) is presented in Figure 4. On the 8th day, with 100% of the clams’ dead, TVC at 7 °C and TVC at 25 °C had increased by less than one and two log cycles, respectively, for both storage conditions. The different TVC change values (log N/N_0_) observed in Figure 4 and described in Table 4 for the same condition tested, G2a (70% O_2_: 30% N_2_, 150 mbar), reinforce the great heterogeneity of the matrix, as indicated in Section 3.1.

Overall, the results indicate that the use of MAP with high oxygen levels increases the saleability of clams, i.e., maintaining the threshold of 95% alive clams, by an additional 1–2 days only. This is very different to what has been reported in the literature for other products, namely for mussels [8,9,33]. This may be related to the metabolism of bivalves in mussels and their living mode, often attached to rocks or other surfaces with stronger currents that can provide flows of well-oxygenated water or be out of the water for long low-tide periods, while clams are always submerged in water, sand or muddy substrates. This may lead to clams being more fragile, whereas mussels are more robust and stand for a much longer time after being harvested [34].

## 4. Conclusions

This study highlighted the critical importance of optimising packaging and storage parameters to extend the shelf-life of live *V. corrugata* clams.

The survival percentage and sensory analysis results indicate that clams stored at intermediate temperature, 5 °C to 8 °C, had the longest time to maintain the threshold of 95% of alive individuals. While this intermediate range benefits the survival of clams at the threshold for sellability, a lower temperature benefits the delay of degradation and slows down the dying rate after the threshold. Storage at 12 °C showed the worst results. This is relevant information for stocking and distributing the product. In addition, it is important to keep the shells closed during storage and tight packaging that provides confinement is essential for this product.

The tests with modified atmosphere packaging showed that the presence of CO_2_ results in the early mortality of the clams. Although the literature contains many articles on the application of a modified atmosphere with a high concentration of oxygen with excellent results for other bivalves, particularly mussels, when applied to this species of clams, this strategy was shown to prolong life by only 1–2 days. This additional shelf-life may not be enough to justify the investment in equipment and the additional operational costs. The selection of packaging materials and conditions is vital to prolonging the shelf-life of *V. corrugata* but always bear in mind that this species is more sensitive than other bivalves and requires special attention to temperature management, the confinement of the clams and the atmospheric composition, with no great industrial advantage from the application of modified atmosphere packaging while alive.

## Figures and Tables

**Table 1:** MAP conditions with vacuum and gas flushing pressure and composition of gases (%).

MAP Conditions	Vacuum (mbar)	Gas Flushing Pressure (mbar)	Composition of Gases (%)		
			O_2_	CO_2_	N_2_
G1	50	150	70	30	-
G2a		150	70	-	30
G2b		120			
G3		150	90	-	10

**Table 2:** Quality scale used in sensory analysis.

Characteristic	Score	
	0	3
Odour	Bad, spoiled, rotten, ammonia-like, sulphurous, putrid	Fresh, sea, characteristic sweet
Appearance	Fresh, bright, pearl-white flesh colour, clear liquid intervalvular	Opaque, parched, white, yellow or brown flesh colour, dark liquid intervalvular

**Table 3:** Initial physical–chemical and microbiological characteristics of each batch of clams: Moisture (%), TVB-N (mg N/100 g) and TVC (log cfu/g).

	Impact of Temperature						Impact of MAP	
	Storage at 3 and 5 °C			Storage at 8 and 12 °C			Effect of CO_2_	Effect of O_2_
	T1	T2	T3	T4	T5	T6	T7	T8
pH	6.5 ± 0.0 ^bc^	6.6 ± 0.1 ^cd^	6.4 ± 0.0 ^a^	6.6 ± 0.0 ^cd^	6.4 ± 0.0 ^ab^	6.5 ± 0.0 ^b^	6.6 ± 0.0 ^d^	6.5 ± 0.0 ^bc^
Moisture	90.0 ± 0.4 ^de^	90.3 ± 0.1 ^e^	88.2 ± 1.3 ^cd^	84.9 ± 0.3 ^b^	82.7 ± 0.1 ^a^	82.7 ± 0.3 ^a^	90.1 ± 0.1 ^de^	87.8 ± 0.0 ^c^
TVB-N	4.4 ± 0.0 ^a^	13.0 ± 0.6 ^bc^	6.7 ± 1.2 ^ab^	3.1 ± 0.6 ^a^	13.8 ± 4.5 ^bc^	19.5 ± 2.7 ^c^	6.4 ± 0.0 ^ab^	<LOD ^a^
TVC 7 °C	2.9 ± 0.1 ^bcd^	4.0 ± 0.0 ^d^	3.7 ± 0.1 ^cd^	3.1 ± 0.3 ^bcd^	2.6 ± 0.1 ^bc^	2.4 ± 0.3 ^b^	<1 ^a^	2.9 ± 0.7 ^bcd^
TVC 25 °C	3.8 ± 0.2 ^abc^	4.1 ± 0.0 ^bc^	4.2 ± 0.2 ^bc^	4.5 ± 0.3 ^c^	3.3 ± 0.0 ^a^	3.6 ± 0.3 ^ab^	3.4 ± 0.1 ^ab^	3.8 ± 0.2 ^abc^

Ti—code of test i; LOD—limit of detection (0.6 mg N/100 g), different letters in the same row indicate significant differences between samples (*p* < 0.05). The results are presented as means ± standard deviation (*n* = 2).

**Table 4:** Physical–chemical and microbiological characteristics of clams under MAP conditions (G1, G2a, G2b) overtime at 3 °C ± 1 °C. Survival percentage (%), liquid exudation (g), moisture (%), TVB-N (mg N/100g) and TVC (log N/N_0_).

	Day 0	Day 3			Day 6		
		G1	G2a	G2b	G1	G2a	G2b
Survival *	100.0 ± 0.0	92.1 ± 2.7 ^a^	97.1 ± 4.3 ^a^	97.9 ± 4.7 ^a^	62.9 ± 14.4 ^A^	94.0 ± 4.2 ^B^	77.7 ± 8.0 ^C^
Exudation *	0.0 ± 0.0	3.5 ± 1.4 ^a^	4.4 ± 1.3 ^a^	3.3 ± 1.3 ^a^	7.2 ± 2.4 ^A^	12.6 ± 8.2 ^A^	9.9 ± 3.3 ^A^
pH **	6.6 ± 0.0	6.8 ± 0.0 ^a^	6.8 ± 0.0 ^a^	6.8 ± 0.0 ^a^	6.7 ± 0.0 ^A^	6.6 ± 0.0 ^A^	6.7 ± 0.1 ^A^
Moisture **	90.1 ± 0.1	89.1 ± 0.4 ^a^	89.5 ± 0.1 ^a^	89.4 ± 0.4 ^a^	87.0 ± 0.8 ^A^	88.0 ± 0.6 ^A^	88.0 ± 0.1 ^A^
TVB-N **	6.4 ± 0.0	8.8 ± 0.0 ^a^	8.8 ± 0.0 ^a^	8.7 ± 0.0 ^a^	11.4 ± 0.0 ^A^	14.9 ± 3.7 ^A,B^	18.1 ± 0.6 ^B^
TVC at 7 °C *	0.0 ± 0.0	2.1 ± 0.8 ^a^	2.2 ± 0.2 ^a^	2.3 ± 0.1 ^a^	2.4 ± 0.2 ^A^	3.1 ± 0.5 ^A,B^	3.3 ± 0.3 ^B^
TVC at 25 °C *	0.0 ± 0.0	0.0 ± 0.2 ^a^	0.1 ± 0.2 ^a^	0.1 ± 0.3 ^a^	1.0 ± 0.4 ^A^	1.2 ± 0.2 ^A^	1.3 ± 0.3 ^A^

Note: G1 (70% O_2_: 30% CO_2_, 150 mbar); G2a (70% O_2_: 30% N_2_, 150 mbar); G2b (70% O_2_: 30% N_2_, 120 mbar). Different letter superscripts in the same row (lowercase for day 3 and uppercase for day 6) indicate significant differences between sample conditions in each day evaluated (*p* < 0.05). The results are presented as means ± standard deviation (* *n* = 5; ** *n* = 2).

**Table 5:** Sensory analysis score of samples under MAP (G1, G2a, G2b) over time 3 °C ± 1 °C.

	Day 0	Day 3			Day 6		
		G1	G2a	G2b	G1	G2a	G2b
Appearance	3.0 ± 0.0	3.0 ± 0.0 ^a^	3.0 ± 0.0 ^a^	3.0 ± 0.0 ^a^	3.0 ± 0.0 ^A^	3.0 ± 0.0 ^A^	3.0 ± 0.0 ^A^
Odour	3.0 ± 0.0	2.5 ± 0.0 ^a^	2.5 ± 0.0 ^a^	3.0 ± 0.0 ^b^	2.0 ± 0.0 ^A^	1.8 ± 0.4 ^A^	2.5 ± 0.0 ^B^

Note: G1 (70% O_2_: 30% CO_2_, 150 mbar); G2a (70% O_2_: 30% N_2_, 150 mbar); G2b (70% O_2_: 30% N_2_, 120 mbar). Different letter superscripts in the same row (lowercase for day 3 and uppercase for day 6) indicate significant differences between sample conditions in each day evaluated (*p* < 0.05). The results are presented as means ± standard deviation (*n* = 5).

## Data Availability

The original contributions presented in the study are included in the article/Supplementary Material, further inquiries can be directed to the corresponding author.

## Supplementary Materials

The following supporting information can be downloaded at https://www.mdpi.com/article/10.3390/foods14091629/s1, Figure S1: Sensory analysis score value of clams stored at different temperatures: (**a**) appearance, (**b**) odour. Limit of acceptability score value below 1. Figure S2: Gas concentration in headspace: (**a**) % oxygen and (**b**) % carbon dioxide during shelf-life evaluation in clams under MAP conditions (G2a, G3). Figure S3: Chemical results: pH (**a**), moisture (**b**) and TVB-N (**c**) of the samples evaluated under different MAP Conditions (G2a, G3) over time. Figure S4: Sensory analysis score value of the samples evaluated under different MAP conditions (G2a, G3) over time: (**a**) appearance, (**b**) odour. Limit of acceptability score value below 1.

## Abbreviations

The following abbreviations are used in this manuscript:

ASP	Amnesic Shellfish Poisoning
CODEX STAN	Codex Alimentarius Standards
CO_2_	Carbon dioxide
DSP	Lipophilic Diarrhetic Shellfish Poisoning
EVOH	Ethylene vinyl alcohol
HCl	Hydrochloric acid
ISO	International Organisation for Standardisation
MAP	Modified atmosphere packaging
NaCl	Sodium chloride
O_2_	Oxygen
OTR	Oxygen transmission rate
PA	Polyamide
PAHS	Polycyclic Aromatic hydrocarbons
PCA	Plate count agar
PE	Polyethylene
PP	Polypropylene
PSP	Paralytic Shellfish Poisoning
RH	Relative humidity
TVB-N	Total volatile basic nitrogen content
TVC	Total viable count
VP	Vacuum packaging
WVTR	Water vapor transmission rate
