# Supplementary material for: Extending the Shelf-Life of Live Clams, Venerupis corrugata—Important Aspects of Current Packaging and Advances in Modified Atmosphere Packaging
Source: Foods. 2025 May 5;14(9):1629. doi: 10.3390/foods14091629 (PMC12072123; doi:10.3390/foods14091629)
Supplement: Supplementary file 1 [file foods-14-01629-s001.zip › foods-3571385-supplementary.pdf]

# Supplementary material

## Extending the shelf-life of live clams, *Venerupis corrugata*. Important aspects of current packaging and advances in modified atmosphere packaging

*Goes C.B., Teixeira, S., Mena, C., Silva, F., Cruz, A., Basílio, I., Hogg, C., Sivertsvik M., Teixeira, P., Poças, F.*

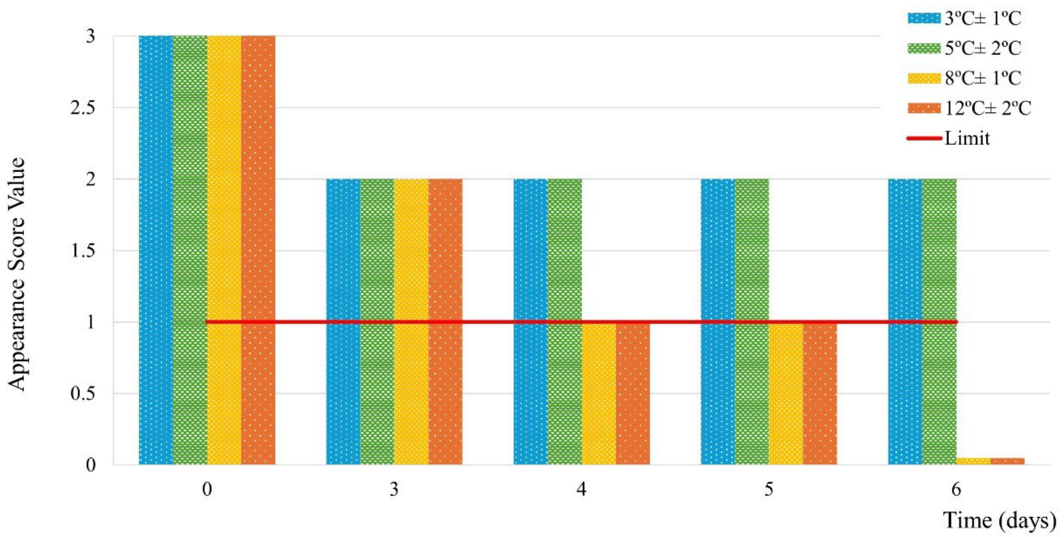


(**a**)

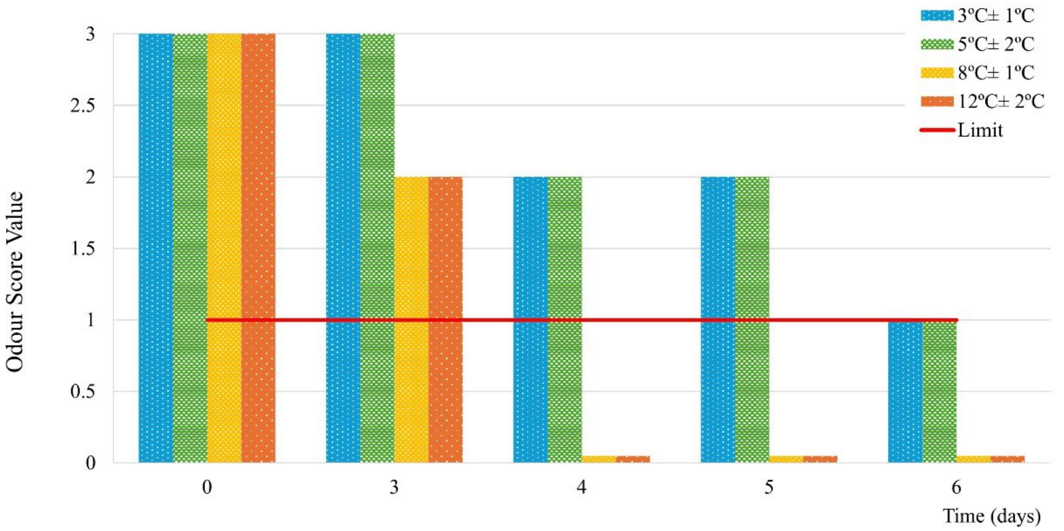


(**b**)

**Figure S1.** Sensory analysis score value of clams stored at different temperatures: (**a**) appearance, (**b**) odour. Limit of acceptability score value below 1.

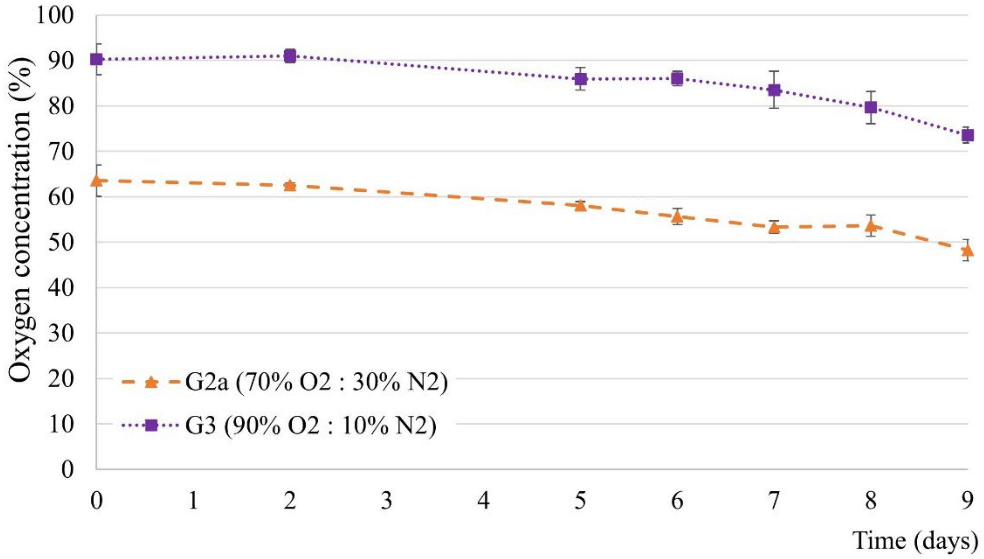


(**a**)

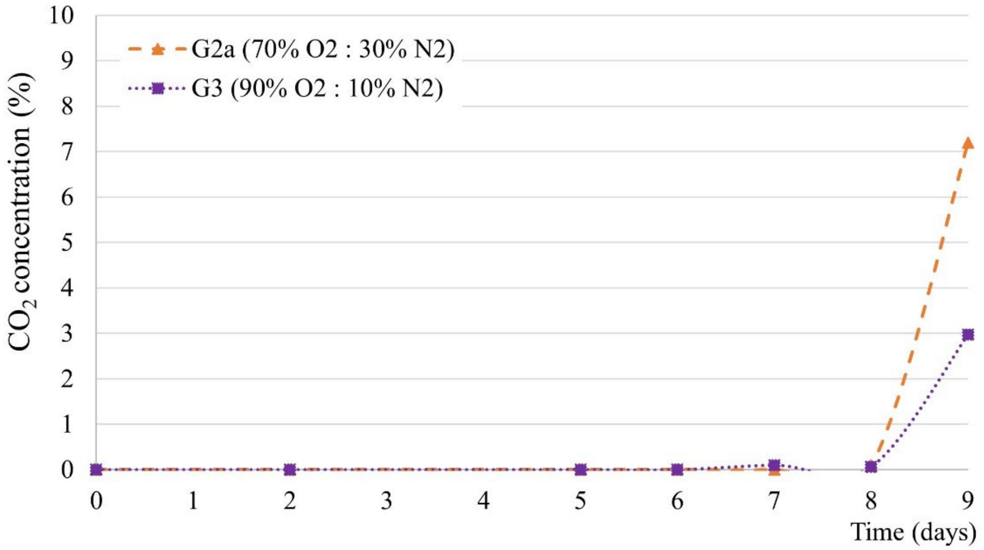


(**b**)

**Figure S2.** Gas concentration in headspace: (**a**) % oxygen and (**b**) % carbon dioxide during shelf-life evaluation in clams under MAP conditions (G2a, G3).

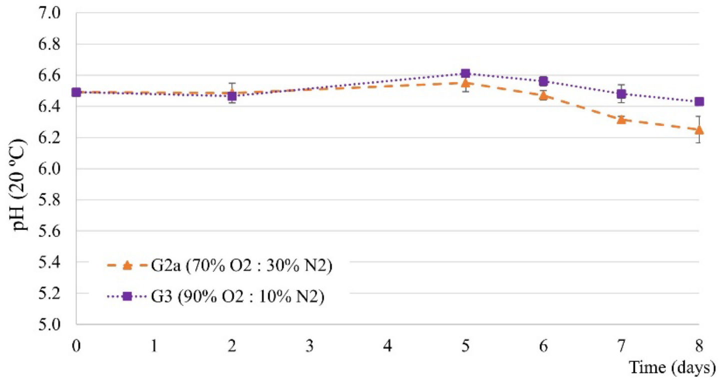


(**a**)

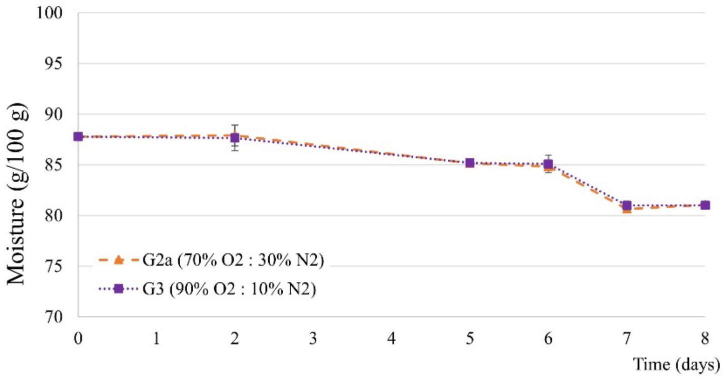


(**b**)

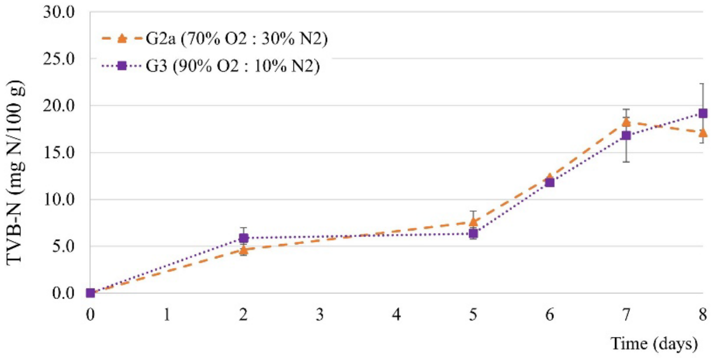


(**c**)

**Figure S3.** Chemical results: pH (**a**), moisture (**b**) and TVB-N (**c**) of the samples evaluated under different MAP Conditions (G2a, G3) over time.

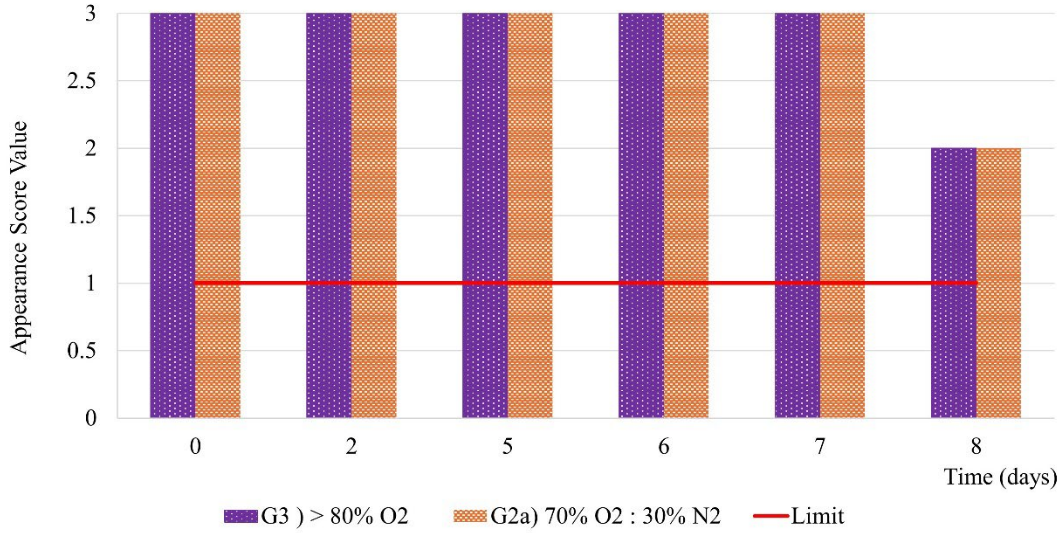


(**a**)

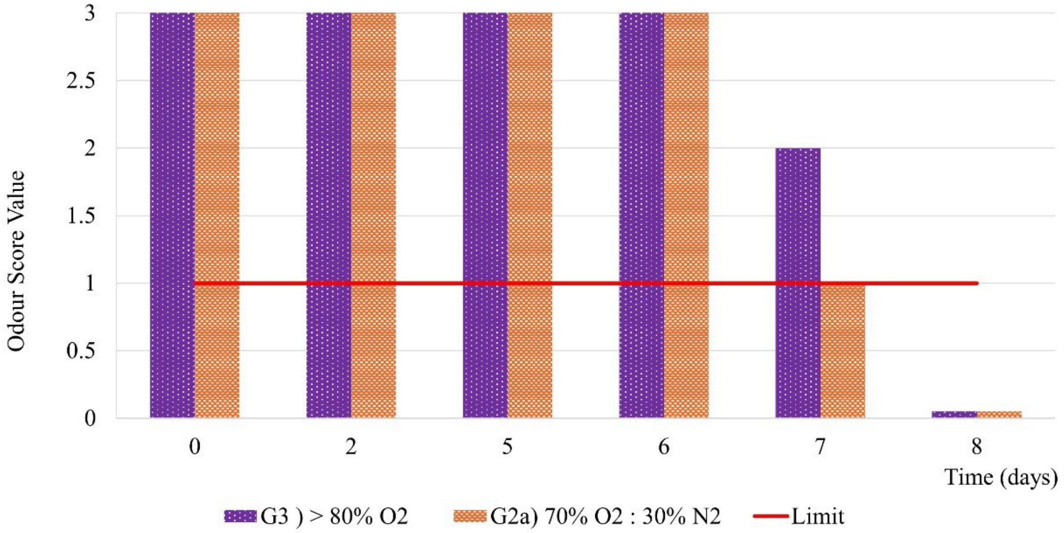


(**b**)

**Figure S4.** Sensory analysis score value of the samples evaluated under different MAP Conditions (G2a, G3) over time: (**a**) appearance, (**b**) odour. Limit of acceptability score value below 1.
